# Burden of opioid use for pain management among adult herpes zoster patients in the US and the potential impact of vaccination

**DOI:** 10.1080/21645515.2022.2040328

**Published:** 2022-04-01

**Authors:** Jean-Etienne Poirrier, Jessica K. DeMartino, Saurabh Nagar, Justin Carrico, Katherine Hicks, Juliana Meyers, Jeffrey Stoddard

**Affiliations:** a GSK, US Health Outcomes and Epidemiology, Philadelphia, PA, USA; b RTI Health Solutions, Health Economics, Research Triangle Park, NC, USA

**Keywords:** Herpes zoster, Herpes zoster vaccination, Medical claims database analysis, Opioid prescription, Painkillers

## Abstract

The goal of this research was to describe treatment patterns, health-care resource utilization, and costs for herpes zoster (HZ)-related pain, and to estimate the potential impact of recombinant zoster vaccine (RZV) on avoided HZ cases and HZ-related pain prescriptions. This retrospective claims database study included patients from commercial, Medicare, and Medicaid plans between 2012 and 2017. Subjects with an HZ episode were assigned to three cohorts: “opioid”, “non-opioid”, and “no-treatment” cohorts. Subjects in the opioid cohort were matched to a non-HZ cohort. The potential impact of RZV vaccination on HZ case avoidance and resulting painkiller prescriptions was modeled. Over 25% of subjects with an HZ episode received opioids. Adjusted health-care costs were approximately double in the opioid cohort versus non-opioid or matched non-HZ cohorts. Postherpetic neuralgia, immunocompromised status, and comorbidities increased the risk for opioid prescription. RZV vaccination was predicted to avoid over 19,000 patients from receiving opioid prescriptions for every 1 million adults aged ≥50 years. HZ-related prescriptions of opioids were common and led to increased health care costs. RZV vaccination may potentially reduce opioid prescriptions through decreasing HZ incidence.

PLAIN LANGUAGE SUMMARY

**What is the context?**

- Herpes zoster or shingles and its complications such as postherpetic neuralgia – a painful condition that affects the nerve fibers and skin – may lead to complex pain that can be addressed using opioids in some patients.
- The recombinant zoster vaccine (RZV) vaccine prevents shingles and, therefore, may reduce the use of opioids and the negative health outcomes and costs associated with it.

**What is new?**

- In this retrospective medical claims study, including patients between 2012 and 2017, we
  - evaluated the receipt of pain medication including opioids in herpes zoster patients, and assessed factors associated with opioid prescription.
  - estimated health care resource utilization and costs associated with opioid use among patients with herpes zoster.
  - assessed the impact of vaccination on opioid prescriptions.
- Among subjects receiving opioids, 78.5% started with a weak opioid dose. Dose escalation was uncommon.
- Postherpetic neuralgia, immunocompromised status, and comorbidities are the main risk factors associated with opioid prescription.
- Health care costs are almost double in patients with herpes zoster receiving opioids compared with patients without an opioid prescription.
- In a population of 1 million adults aged 50 years or older, vaccination with the recombinant zoster vaccine could prevent over 19,000 patients from receiving opioids.

**What is the impact?**

- Prevention of herpes zoster through vaccination may be a highly effective strategy to reduce opioid prescriptions and costs related to pain management in a susceptible population.
- Increasing RZV vaccination coverage in adults aged ≥50 years may further reduce potential opioid prescriptions through a decrease in shingles incidence.

## Introduction

In the United States (US), an estimated 99.5% of the population aged ≥40 years is infected with varicella-zoster virus (VZV). After recovery from infection, VZV remains dormant in a person's body.^1^ Immunosenescence due to aging or immunosuppression due to disease and/or therapy may lead to reactivation of VZV, resulting in herpes zoster (HZ). Without vaccination, approximately 30% of people will develop HZ during their lifetime with the incidence of HZ increasing with age.^1^ HZ leads to a unilateral, painful rash, which typically lasts 7 to 10 days, and complete healing occurs after 2 to 4 weeks.^1^ The pain associated with HZ has been described as “aching, burning, stabbing, or shock-like” and individuals with HZ may also experience “altered sensitivity to touch, pain provoked by trivial stimuli, and unbearable itching”.^2^

A common complication of HZ is postherpetic neuralgia (PHN), which occurs when pain persists ≥90 days after the zoster rash has resolved.^2^ Pain associated with PHN may be mild to severe, and can disrupt all aspects of daily life, including sleep, mood, work, and daily activities. Pain associated with PHN can last for several months and occasionally for several years or more.^3,4^

Given that pain is by far the most debilitating symptom associated with HZ and PHN, adequate pain control is paramount. There are no published recommendations specific for pain associated with HZ, but several treatment guidelines exist for pain due to PHN.^5–8^ Effective treatments include anticonvulsants (e.g., gabapentin or pregabalin), tricyclic antidepressants, and opioids. Opioids were shown to be effective in the treatment of PHN-related pain and are recommended as third-line option; however, their use is limited by the development of dependence and analgesic tolerance.^5,6,9^ In the context of the current opioid crisis in the US, different strategies have been put in place to reduce opioid misuse.^10^

Several US-based studies have evaluated opioid use in patients with PHN and factors that affect opioid use in HZ patients. In a retrospective claims database analysis, 20% of patients diagnosed with PHN and treated with gabapentin as first-line therapy switched to an opioid while 21% augmented treatment with an opioid.^11^ Another retrospective claims database analysis showed that 5% of opioid-naïve patients were treated directly with opioids after a PHN diagnosis and that 46% switched to an opioid after initially trying a non-opioid medication.^12^ Finally, a retrospective electronic health record analysis showed that 35% of patients treated for PHN received opioids; among patients who received opioids, 85% received them as first-line treatment.^13^ Suaya et al. showed that adult patients with diabetes mellitus who developed HZ more frequently received an opioid prescription versus controls.^14^ Li et al. showed that immunocompromised patients with HZ were more likely to have an opioid prescription versus controls without HZ.^15^

From a prevention perspective, vaccination against HZ may indirectly curb the number of opioid and other pain medication prescriptions by lowering HZ incidence in people susceptible to HZ. Recombinant zoster vaccine (RZV) is a two-dose recombinant subunit vaccine, approved by the Food and Drug Administration in October 2017 for the prevention of HZ in individuals aged ≥50 years.^16^ Post-hoc analyses of a phase 3 clinical study data revealed that the use and duration of pain medication associated with HZ was reduced in patients receiving RZV compared with the placebo group.^17,18^

Published data are limited to specific populations and/or lack a comprehensive view of the overall population affected by HZ regardless of health care plans, geographic location, and clinical presentation. To understand the value of HZ prevention in the US, a comprehensive picture of HZ and PHN-related pain medication utilization is required.

The primary objectives of this study were to evaluate receipt of pain-related medications (e.g. opioids, benzodiazepines) among patients with HZ, compare patient demographics and treatment patterns in patients with HZ receiving opioids to those receiving other pain medication, and assess factors associated with opioid prescription.

The secondary objective was to evaluate the health care resource utilization (HCRU) and costs associated with opioid use among patients with HZ. The third objective was to estimate the number of patients needed to vaccinate with RZV to avoid one opioid or other pain medication prescription.

## Methods

This was a retrospective medical claims database study to examine current treatment patterns for HZ-related pain as well as associated treatment costs and HCRU. Data were sourced from the IBM Watson Health Analytics' MarketScan Suite and included claims from the Commercial Claims and Encounters (“Commercial”) database, the Medicare Supplemental (Medicare) database and the Medicaid Multistate (Medicaid) database (IBM Watson Health Analytics).^19^ The MarketScan databases contain information on over 273 million unique patients since 1995, with nearly 40 million patients included in the most recent calendar year of data available. The Commercial database contains data from active employees, early retirees, Consolidated Omnibus Budget Reconciliation Act continuees, and dependents insured by employer-sponsored plans. The Medicare database includes Medicare-eligible retirees with employer-sponsored Medicare Supplemental plans. The Medicaid database contains approximately seven million Medicaid enrollees from multiple states.

The patient identification window for the Commercial/Medicare databases was from 1 February 2014, to 31 March 2017, and from 1 July 2012, to 31 December 2015 for the Medicaid database.

Data used in this study were de-identified and fully compliant with the Health Insurance Portability and Accountability Act. The study did not meet the definition of research in human subjects.

### Subjects and study cohorts

Two overall study populations were evaluated in this analysis: those with an episode of HZ (termed the overall HZ population) and those without record of HZ at any time point. Subjects who had received HZ vaccination during the observation period were excluded.

Subjects were eligible for inclusion in the overall HZ population if they were aged ≥18 years, and had a primary or secondary diagnosis of HZ (International Classification of Diseases, 9^th^ Revision, Clinical Modification [ICD-9-CM] diagnosis code 053.xx and International Classification of Diseases, 10^th^ Revision, Clinical Modification [ICD-10-CM] diagnosis code B02.xx). The first observed HZ diagnosis defined the “HZ index date.” Subjects were required to have continuous health plan enrollment for at least 6 months before and for at least 12 months after the HZ index date. Subjects with a diagnosis of postherpetic trigeminal neuralgia or postherpetic polyneuropathy on the HZ index date were excluded since these diagnoses were indicative of continuation of care for HZ rather than a first HZ episode. Subjects with a history of opioid disorder or who received a HZ vaccine were excluded.

The overall HZ population was divided into three mutually exclusive cohorts:

- Opioid HZ cohort: subjects who had at least one claim for an opioid medication during the 12 months after the HZ index date and no claim of opioid or non-opioid pain medication or HZ/PHN-related medication 6 months prior to the HZ index date.
- Non-opioid HZ cohort: subjects who had at least one claim for a non-opioid pain medication or an HZ-/PHN-related medication and no opioid claim during the 12 months after the HZ index date. Subjects with receipt of opioid or non-opioid pain medication or HZ/PHN-related medication in the 6 months prior to the HZ index date were excluded.
- No-treatment HZ cohort: subjects who had no claim for any pain medication or HZ-/PHN-related treatment during the 12 months after the index date.

A list of the HZ/PHN-related medications used in the analysis is provided in Table 1.

**Table 1.** List of HZ-/PHN-related medications and pain medications

Class	Generic names
HZ-/PHN-related medications	
Antivirals (oral and intravenous)	Acyclovir, valacyclovir, famciclovir
Non-opioid pain-related medications	
Analgesic agents	Acetaminophen, nonsteroidal anti-inflammatory drugs
Anticonvulsants	Gabapentin, pregabalin
Benzodiazepines	Adinazolam, alprazolam, chlordiazepoxide, climazolam, clobazam, clonazepam, clorazepate, diazepam, estazolam, flurazepam, halazepam, loprazolam, lorazepam, lormetazepam, midazolam, nimetazepam, nitrazepam, oxazepam, prazepam, temazepam, triazolam, quazepam
Oral corticosteroids	Prednisone, hydrocortisone, prednisolone, methylprednisolone, triamcinolone, paramethasone, dexamethasone, betamethasone
Tricyclic antidepressants	Amitriptyline, desipramine, nortriptyline
Topical pain relievers	Capsaicin, lidocaine
Muscle relaxants	Flexeril (cyclobenzaprine)
Other medications	Memantine
Opioids	
Weak opioids	Codeine, dihydrocodeine, hydrocodone, pentazocine, propoxyphene, tramadol
Strong opioids	Buprenorphine, diamorphine, fentanyl, hydromorphone, meperidine, methadone, morphine, oxymorphone, oxycodone

HZ: herpes zoster; PHN: postherpetic neuralgia.

Source: Fashner and Bell (2011).^20^

Subjects were eligible for inclusion in a non-HZ population (non-HZ cohort) if they were aged ≥18 years, did not have a diagnosis of HZ at any point in time, and had at least 18 months of continuous health plan enrollment. Subjects in the opioid HZ cohort, were matched in a 1:5 ratio to subjects in the non-HZ cohort, using direct covariate matching. Variables used during the matching procedure were age, sex, payer type (commercial vs Medicaid), comorbidity index, and immunocompromised status.

### Endpoints

Demographics and clinical characteristics were analyzed for all subjects by cohort at the HZ index date. Each patient's Charlson Comorbidity Index (CCI) score was calculated during the 6-month period before the HZ index date. The CCI score takes into account 20 categories of comorbidities, as defined by ICD-9-CM and ICD-10-CM diagnosis and procedure codes, with associated weights corresponding to the severity of the comorbid conditions of interest.^21,22^ The number of subjects developing PHN or other HZ-related complications at any point in time after the HZ index date (and regardless of the timing of the first observed receipt of opioid/non-opioid treatment) were identified using methodology described previously.^23,24^

Treatment patterns were analyzed (e.g., type and class, start date, and treatment duration) and factors associated with receipt of opioids were analyzed by multivariate regression analysis.

HCRU and costs associated with opioid use were calculated by time period and care location for all cohorts and incremental HCRU and costs were evaluated comparing the opioid HZ cohort to the non-opioid HZ cohort and the opioid HZ cohort to the matched non-HZ cohort.

### Statistical analysis

Descriptive statistics were used to describe demographics, clinical characteristics, treatment patterns, HCRU, and costs. Counts and percentages were provided for categorical data; mean, median, SD were calculated for continuous variables.

A logistic regression model was developed to determine factors associated with receipt of at least one opioid prescription at any point during the 1-year follow-up period. Covariates included in the model were age, sex, health plan, geographic region, race, year of HZ index date, CCI score, immunocompromised status, and presence of HZ complications, including PHN.

Multivariable regression models were used to estimate adjusted incremental costs associated with opioid use among subjects with HZ. To handle the skewed nature of the cost distribution common in claims data, generalized linear models with a log link function and gamma distribution for the error term were applied.^25,26^

### Impact of HZ vaccination on prescription of opioids and other pain medication

An Excel-based calculator was developed to apply simple calculations to estimates from the published literature, outcomes of the claims database analysis, and outcomes from a previously published cost-effectiveness model^27,28^ to assess the potential impact of RZV vaccination  on opioid and other pain medication prescription outcomes compared with no vaccination over a lifetime horizon in patients aged ≥50 years.^29^

The calculator was parametrized using estimates of painkiller prescription rates (opioids, benzodiazepines, and other painkillers) among HZ cases based on the analysis of claims databases described above, with results weighted across health plans to be more representative of the general US population. Parameters for HZ case avoidance attributable to RZV vaccination, assuming a coverage of 65%, were obtained from a previously published cost-effectiveness model.^27,28^

Outcomes included the number of opioid and non-opioid prescriptions avoided, number needed to vaccinate to prevent one painkiller prescription, and number of opioid-related adverse events avoided (long-term opioid use, emergency department [ED] visit, death).^30^

## Results

**Figure 1.**
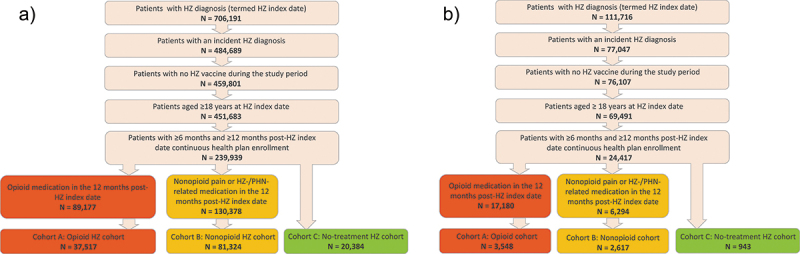
Flow chart of selection of subjects from (a) commercial/Medicare databases and (b) Medicaid database.

HZ: herpes zoster; PHN: postherpetic neuralgia.

Overall, 139,225 and 7,108 subjects had a diagnosis of HZ in the commercial/Medicare and Medicaid databases, respectively, and no HZ-related pain medication in the 6 months preceding the HZ episode. Among these subjects, 26.9% and 49.9% had an opioid prescription claim in the commercial/Medicare and Medicaid population, respectively (Figure 1).

### Demographic and clinical characteristics

Demographic characteristics in the unmatched populations were similar across the opioid, non-opioid, and no-treatment cohorts. Mean age ranged from 50.8 to 52.2 years and there were more female (57.7% − 58.8%) than male (41.2% − 42.3%) subjects.

A total of 39,299 subjects from the combined opioid HZ cohort were matched to 196,495 subjects in the non-HZ cohort (Table 2). Most subjects (91.5% in combined HZ and matched non-HZ cohort) were covered by commercial payers or Medicare. Consistent with the geographic distribution of the MarketScan database, the largest percentage of patients were from the South.

**Table 2.** Demographic and clinical characteristics - matched populations

Characteristic, Statistics	Opioid HZ cohort, N = 39,299	Non-HZ cohort, N = 196,495
Age, mean (SD)	51.0 (15.8)	50.2 (16.7)
Male, %	41.1%	41.1%
Commercial or Medicare payer, %	91.5%	91.5%
Health plan type, %		
Health maintenance organization	14.8%	13.1%
Preferred provider organization	51.3%	50.4%
Point of service	6.3%	6.2%
Other	26.6%	25.4%
Missing/unknown	1.0%	4.9%
Geographic region, %		
Northeast	14.0%	16.8%
North central	21.0%	20.5%
South	40.9%	38.1%
West	15.3%	13.8%
Unavailable (Medicaid subjects)	8.5%	10.5%
CCI score, mean (SD)^a^	0.8 (1.4)	0.8 (1.4)
Common CCI comorbidities, %		
Hypertension	25.2%	23.7%
Diabetes	10.2%	10.0%
Chronic pulmonary disease	6.2%	6.6%
Patients with an immunocompromised diagnosis,	12.4%	12.4%
Common immunocompromised diagnoses^c^		
Cancer	5.9%	4.9%
Solid organ transplant	3.9%	4.5%
Patients with an HZ-related complication	44.7%	NA
Types of HZ-related complications observed		
PHN	42.7%	NA
Cutaneous complications	2.2%	NA
Neurological complications (excluding PHN)	0.4%	NA
Ophthalmic complications	4.8%	NA

CCI: Charlson Comorbidity Index; HZ: herpes zoster; PHN: postherpetic neuralgia; SD: standard deviation.

^a^The CCI was calculated based on data recorded during the 6 months prior to the HZ index date.^21^

Subjects with an HZ episode showed a similar pattern of comorbidities across cohorts. The number of subjects with PHN was highest in the opioid HZ cohort (43.0%), followed by the non-opioid HZ cohort (27.6%), and the no-treatmentHZ cohort (12.7%). After matching of subjects in the opioid HZ and non-HZ cohorts, clinical characteristics not related to HZ were comparable between the two cohorts (Table 2).

### Treatment patterns

In the opioid cohort, the most common first-line treatments received after the HZ index date were valacyclovir alone (19.6%), followed by valacyclovir plus hydrocodone (11.3%), acyclovir alone (7.0%), acyclovir plus hydrocodone (5.5%), and valacyclovir plus prednisone (5.4%). In the non-opioid cohort, a higher percentage of subjects received valacyclovir as first line treatment (47.5%), followed by acyclovir alone (14.5%), valacyclovir plus prednisone (8.7%), and famciclovir alone (5.8%).

Among subjects receiving opioids, 78.5% started with a weak opioid (codeine or hydrocodone), while approximately 20% of subjects received a strong opioid, mostly oxycodone.

Median time from HZ index date to first opioid prescription was 3 days (range: 1–366 days). More than half of subjects (52.3%) received other treatments (mostly antivirals) prior to starting opioid treatment. Prior treatment with analgesics (6.7%), anticonvulsants (8.0%) and oral corticosteroids (7.0%) was uncommon. Most patients (56%) received opioids concomitantly with another treatment, usually an antiviral. Very few subjects initiated opioid treatment with a benzodiazepine (1.6%), though 7.3% of subjects received benzodiazepine prescriptions after starting opioid therapy. Prescribing other HZ/PHN-related agents after the start of opioid therapy was common (45.8%). The median opioid supply was 5.0 days at a median morphine equivalent dose of 5.0 mg. Dose escalation was observed in 10.9% of subjects and only 6.0% of subjects switched from a weak to a strong opioid. On average, subjects had 1.6 opioid prescriptions after the HZ index date, with more than two-thirds of patients having only a single prescription during follow-up. The percentage of patients with opioid prescriptions after the first month from the HZ index date remained relatively constant from months 2 to 12 (range: 6.9% to 7.8%) (Table 3).

**Table 3.** Opioid dosage patterns

Opioid HZ cohort		
Characteristic, statistic	41,065	100.0%
Days opioid medication supplied		
Mean (SD)	5.9	5.3
Median	5	
Minimum, maximum	1.0	90
Opioid dose at initiation (morphine milligram equivalent in mg)		
Mean (SD)	6.2	2.1
Median	5	
Minimum, maximum	1.8	60
Number of opioid prescriptions during the postindex period		
Mean (SD)	1.6	1.6
Median	1	
Minimum, maximum	1	35
Number of opioid prescriptions filled during the postindex period		
1	28,070	68.4%
2	7,676	18.7%
3	2,622	6.4%
4 +	2,697	6.6%
Days from HZ index date to first observed opioid prescription		
Mean (SD)	75.3	109.0
Median	3.0	
Minimum, maximum	1.0	366.0
Patients filling a prescription for an opioid during each month of the follow-up period (N, %)		
Month 1	25,203	61.4%
Month 2	3,194	7.8%
Month 3	2,915	7.1%
Month 4	2,935	7.1%
Month 5	2,836	6.9%
Month 6	2,921	7.1%
Month 7	2,816	6.9%
Month 8	2,815	6.9%
Month 9	2,953	7.2%
Month 10	2,872	7.0%
Month 11	2,883	7.0%
Month 12	3,058	7.4%
Opioid dose escalation during the postindex period		
No	36,609	89.1%
Yes	4,456	10.9%
Patients switched from weak to strong opioids during the postindex period		
No	38,596	94.0%
Yes	2,469	6.0%

HZ: herpes zoster; SD: standard deviation.

### Factors associated with receipt of opioid therapy

Among the variables included in the logistic regression model, PHN was the strongest predictor of receipt of opioid therapy (odds ratio [OR]: 2.35 in the commercial population, 2.36 in the Medicare population and 2.65 in the Medicaid population; P < 0.0001) (Table 4). The OR for receiving opioid therapy decreased in later calendar years, suggesting a trend of fewer opioid prescriptions over time. Other factors associated with opioid therapy included immunocompromised status, presence of comorbidities and geographic region other than Northeast (commercial and Medicare databases only).

**Table 4.** Factors associated with receipt of opioid medication from multivariable logistic regression analysis – commercial database

Factor	Odds Ratio (95% CI)	*P*
Presence of HZ complications (versus no complications)		
PHN (versus no PHN)	2.35 (2.28; 2.42)	<0.01
Cutaneous complications	0.95 (0.87; 1.05)	0.34
Neurological complications (excluding PHN)	1.05 (0.84; 1.30)	0.68
Ophthalmic complications	0.82 (0.77; 0.88)	<0.01
Age on HZ index date (versus <50 years)		
50–54 years	1.05 (1.01; 1.09)	0.02
55–59 years	1.06 (1.03; 1.10)	<0.01
60–64 years	1.04 (1.00; 1.09)	0.04
Males (versus females)	1.01 (0.98; 1.04)	0.55
Health plan type (versus health maintenance organization		
Preferred provider organization	1.06 (1.01; 1.10)	0.01
Point of service	1.13 (1.06; 1.21)	<0.01
Other	1.06 (1.01; 1.12)	0.02
Geographic region (versus Northeast)		
North Central	1.46 (1.40; 1.53)	<0.01
South	1.71 (1.64; 1.78)	<0.01
West	1.57 (1.50; 1.65)	<0.01
Year of HZ index date (versus 2014)		
2015	0.93 (0.90; 0.96)	<0.01
2016	0.83 (0.80; 0.86)	<0.01
2017	0.74 (0.70; 0.79)	<0.01
CCI score (versus CCI score = 0)		
CCI score 1	1.14 (1.10; 1.18)	<0.01
CCI score 2	1.26 (1.19; 1.33)	<0.01
CCI score 3	1.24 (1.14; 1.35)	<0.01
CCI score ≥4	1.38 (1.28; 1.50)	<0.01
Presence of immunocompromised status (versus no immunocompromised diagnosis)	1.21 (1.15; 1.27)	<0.01

CCI: Charlson Comorbidity Index; CI: confidence interval; HZ: herpes zoster; P, P-value; PHN: postherpetic neuralgia.

### Health care resource utilization and costs

**Figure 2.**
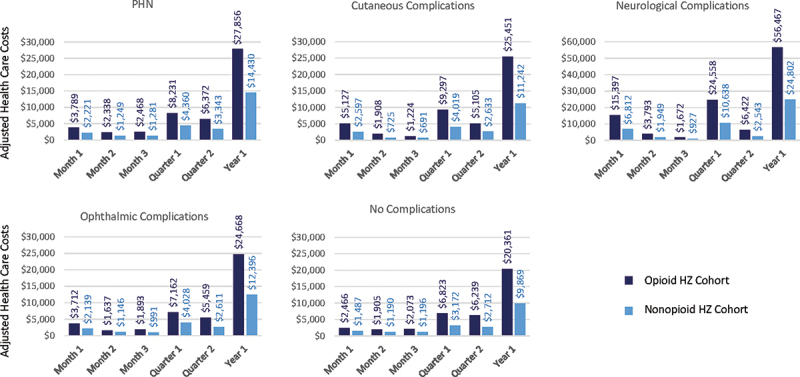
Adjusted health care costs by time from HZ Index date and by type of complication.

HZ: herpes zoster; PHN: postherpetic neuralgia.

In the first month after the HZ index date, the adjusted health care costs estimated by the model were $2,545 and $1,317, in the opioid HZ and non-opioid HZ cohorts, respectively, while in the first year after the HZ index date, adjusted health care costs were $23,599 and $9,937 in the opioid and non-opioid HZ cohorts, respectively (Table 5). Similar trends were observed between the opioid and matched non-HZ cohorts (Table 5). Costs slightly decreased in the first month after the HZ index date, but the incremental cost increase between the opioid and non-opioid HZ cohorts remained constant over time. HZ-related complications, including PHN, resulted in an increase in adjusted health care costs, especially in the case of neurological complications ($56,467 and $20,361 for neurological versus no complications, respectively during first year after the HZ index date) (Figure 2). Adjusted health care costs were highest in the Medicare sample and lowest in subjects covered by Medicaid (Table 5).

**Table 5.** Adjusted health care costs by time from HZ index date and by payer

*Opioid HZ cohort vs non-opioid HZ cohorts*												
	Overall			Commercial			Medicare			Medicaid		
Time period after the index date	Opioid HZ cohort	Non-opioid HZ cohort	Difference	Opioid HZ cohort	Non-opioid HZ cohort	Difference	Opioid HZ cohort	Non-opioid HZ cohort	Difference	Opioid HZ cohort	Non-opioid HZ cohort	Difference
Month 1	$2,545	$1,317	$1,228	$2,663	$1,375	$1,288	$3,285	$1,822	$1,463	$1,383	$1,107	$276
Month 2	$2,208	$1,104	$1,104	$2,443	$1,204	$1,239	$2,478	$1,473	$1,005	$1,392	$1,227	$165
Month 3	$2,235	$1,078	$1,157	$2,464	$1,183	$1,281	$2,692	$1,477	$1,215	$1,894	$1,467	$427
Quarter 1	$6,081	$2,845	$3,236	$6,759	$3,136	$3,623	$7,815	$4,294	$3,521	$4,145	$3,214	$931
Quarter 2	$5,899	$2,667	$3,232	$6,134	$2,748	$3,386	$6,823	$3,713	$3,110	$4,439	$3,192	$1,247
Year 1	$23,599	$9,937	$13,662	$25,389	$10,650	$14,739	$27,862	$15,699	$12,163	$20,048	$13,730	$6,318

*Opioid HZ cohort vs matched non-HZ cohort*												
	Overall			Commercial			Medicare			Medicaid		
Time period after the index date	Opioid HZ cohort—matched	Matched non-HZ cohort	Difference	Opioid HZ cohort—matched	Matched non-HZ cohort	Difference	Opioid HZ cohort—matched	Matched non-HZ cohort	Difference	Opioid HZ cohort—matched	Matched non-HZ cohort	Difference
Month 1	$2,329	$1,118	$1,211	$2,298	$1,287	$1,011	$3,048	$1,645	$1,403	$1,378	$1,023	$355
Month 2	$2,178	$1,028	$1,150	$2,381	$1,029	$1,352	$2,356	$1,389	$967	$1,287	$1,185	$102
Month 3	$2,089	$987	$1,102	$2,310	$1,018	$1,292	$2,519	$1,369	$1,150	$1,890	$1,329	$561
Quarter 1	$5,812	$2,516	$3,296	$6,429	$2,891	$3,538	$7,618	$4,109	$3,509	$4,099	$3,105	$994
Quarter 2	$5,690	$2,128	$3,562	$5,981	$2,579	$3,402	$6,694	$3,683	$3,011	$4,328	$3,078	$1,250
Year 1	$21,892	$9,104	$12,788	$24,971	$9,872	$15,099	$26,955	$15,088	$11,867	$19,873	$13,029	$6,844

HZ: herpes zoster.

In general, HCRU was higher in the opioid HZ cohort compared with the non-opioid HZ cohort, with more subjects reporting inpatients stays (2.4% vs 0.8%), ED visits (22.1% vs 9.5%), and outpatient visits (25.6% vs 15.3%) in the first month after the HZ index date. More than 80% of subjects had at least one physician office visit and at least one pharmacy prescription regardless of cohort (Table 6). The relative contribution of cost items to total costs was different between the opioid and non-opioid HZ cohorts, reflecting differences in HCRU: in the opioid HZ cohort in the first month after the HZ index date, the largest cost drivers were inpatient stays (32.9%), outpatient hospital visits (19.1%), and pharmacy (15.5%) compared with physician office visit (25.1%), pharmacy (24.8%), and inpatient visits (18.7%) in the non-opioid cohort.

**Table 6.** Health care resource utilization and costs, by cohort (month 1 after the index date)

	Unmatched patients				Matched patients			
Characteristic	Opioid HZ cohort (N = 41,065)		Non-opioid HZ cohort (N = 83,941)		Opioid HZ cohort—matched (N = 39,299)		Non-HZ cohort—matched (N = 196,495)	
**Inpatient services**								
≥1 hospital admission (N, %)	992	2.4%	679	0.8%	875	2.2%	1,589	0.8%
Number of admissions								
Mean (SD)	0.0	0.2	0.0	0.1	0.0	0.2	0.0	0.1
Median	0.0		0.0		0.0		0.0	
Range (minimum, maximum)	0.0	4.0	0.0	5.0	0.0	4.0	0.0	5.0
Total hospital days								
Mean (SD)	4.7	5.3	4.5	5.7	4.5	4.9	4.4	6.0
Median	3.0		3.0		3.0		3.0	
Range (minimum, maximum)	1.0	64.0	1.0	92.0	1.0	64.0	1.0	96.0
Inpatient costs								
Mean (SD)	$603	$7,053	$176	$3,619	$539	$6,646	$187	$5,180
Median	$0		$0		$0		$0	
Range (minimum, maximum)	$0	$561,232	$0	$436,417	$0	$561,232	$0	$1,425,620
**ED visits**								
≥1 ED visit (N, %)	9,065	22.1%	7,941	9.5%	8,601	21.9%	4,729	2.4%
Number of unique days with an ED visit								
Mean (SD)	0.3	0.6	0.1	0.3	0.3	0.6	0.0	0.2
Median	0.0		0.0		0.0		0.0	
Range (minimum, maximum)	0.0	14.0	0.0	6.0	0.0	14.0	0.0	12.0
ED costs								
Mean (SD)	$176	$788	$64	$504	$173	$770	$25	$354
Median	$0		$0		$0		$0	
Range (minimum, maximum)	$0	$37,261	$0	$33,902	$0	$37,261	$0	$44,183
**Outpatient hospital visits**								
≥1 outpatient hospital visits (N, %)	10,506	25.6%	12,852	15.3%	9,848	25.1%	21,025	10.7%
Number of unique days with an outpatient hospital visit								
Mean (SD)	0.4	0.9	0.2	0.7	0.4	0.9	0.2	0.7
Median	0.0		0.0		0.0		0.0	
Range (minimum, maximum)	0.0	26.0	0.0	23.0	0.0	26.0	0.0	31.0
Outpatient hospital costs								
Mean (SD)	$351	$2,372	$141	$1,396	$336	$2,305	$190	$2,109
Median	$0		$0		$0		$0	
Range (minimum, maximum)	$0	$169,758	$0	$156,599	$0	$169,758	$0	$235,502
**Physician office visits**								
≥1 physician office visit (N, %)	35,199	85.7%	74,864	89.2%	33,681	85.7%	69,192	35.2%
Number of unique days with a physician office visits								
Mean (SD)	1.8	1.6	1.7	1.4	1.8	1.6	0.7	1.3
Median	1.0		1.0		1.0		0.0	
Range (minimum, maximum)	0.0	31.0	0.0	31.0	0.0	31.0	0.0	31.0
Physician office visit costs								
Mean (SD)	$274	$863	$236	$523	$271	$860	$123	$666
Median	$147		$135		$145		$0	
Range (minimum, maximum)	$0	$69,031	$0	$35,994	$0	$69,031	$0	$134,610
**Pharmacy**								
≥1 prescription (N, %)	39,861	97.1%	82,374	98.1%	38,147	97.1%	95,425	48.6%
Number of prescription claims								
Mean (SD)	4.1	2.9	2.8	2.1	4.0	2.9	1.5	2.4
Median	3.0		2.0		3.0		0.0	
Range (minimum, maximum)	0.0	131.0	0.0	54.0	0.0	131.0	0.0	50.0
Pharmacy costs								
Mean (SD)	$285	$1,029	$233	$1,088	$275	$998	$164	$953
Median	$61		$45		$60		$0	
Range (minimum, maximum)	$0	$37,980	$0	$102,224	$0	$37,980	$0	$95,778
**Other medical encounters**								
≥1 other medical encounter (N, %)	11,430	27.8%	20,561	24.5%	10,817	27.5%	24,040	12.2%
Number of unique days with other medical encounters								
Mean (SD)	0.5	1.4	0.4	1.3	0.4	1.4	0.2	1.2
Median	0.0		0.0		0.0		0.0	
Range (minimum, maximum)	0.0	31.0	0.0	31.0	0.0	31.0	0.0	31.0
Other medical encounter costs								
Mean (SD)	$146	$1,557	$88	$1,078	$137	$1,532	$85	$1,140
Median	$0		$0		$0		$0	
Range (minimum, maximum)	$0	$136,228	$0	$140,764	$0	$136,228	$0	$144,350
**Total health care costs**								
Mean (SD)	$1,834	$8,243	$939	$4,565	$1,731	$7,845	$775	$6,037
Median	$419		$294		$409		$39	
Range (minimum, maximum)	$0	$565,076	$0	$438,574	$0	$565,076	$0	$1,441,256

ED: emergency department; HZ: herpes zoster; SD: standard deviation.

HCRU in the opioid HZ cohort was higher compared with the matched non-HZ cohort. In the opioid HZ cohort, more subjects had inpatient stays, and ED, and outpatient visits compared with the matched non-HZ cohort; subjects in the opioid HZ cohort were more likely to have at least one physician office visit compared with subjects in the matched non-HZ cohort (85.7% vs 35.2%).

**Figure 3.**
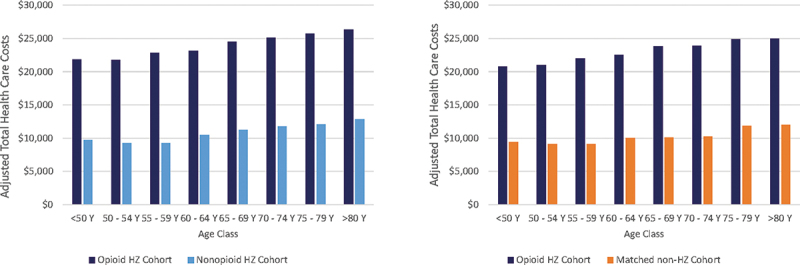
Adjusted health care costs in the 1-year after the HZ index date, by age.

In all cohorts, adjusted health care costs increased slightly with increasing age (Figure 3).

Left: non-matched cohorts HZ-population; Right: matched cohorts, matched opioid cohort and matched non-HZ cohort.

HZ: herpes zoster; Y: years.

### Potential impact of RZV vaccination

The calculator to assess the potential impact of RZV vaccination on pain medication outcomes estimated that, depending on age-group, between 24.4% and 28.0% of HZ cases would lead to at least one prescription of opioids. Between 4.5% and 6.5% of subjects would receive benzodiazepines and between 8.6% and 19.6% other painkillers for HZ/PHN-related pain depending on the age-group.

**Figure 4.**
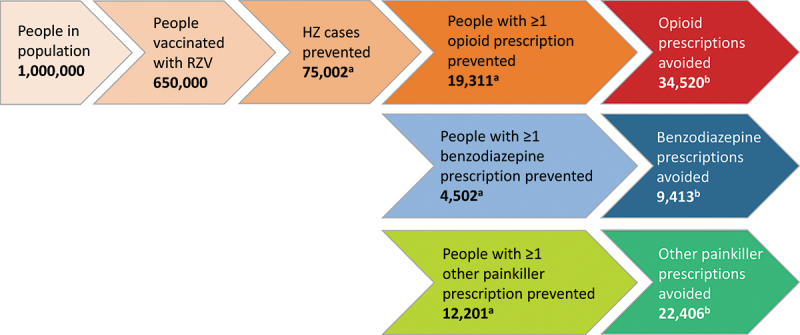
**HZ-related prescriptions avoided with RZV vaccination in a population of 1 million adults aged ≥50 years** .

^a^Calculated based on the size of the population vaccinated with RZV and the NNV to avoid one HZ case from RZV cost-effectiveness model.^41^

^b^Calculated based on number of HZ cases prevented and painkiller prescription rates.

HZ: herpes zoster; NNV: number needed to vaccinate; RZV: recombinant zoster vaccine

Assuming a population of 1 million people aged ≥50 years and vaccination coverage of 65%, RZV vaccination could avoid 75,002 HZ cases over a lifetime horizon.^28^ Overall, 19,311 subjects could be spared from HZ-related opioid prescriptions and 4,502 and 12,201 subjects could forego HZ-related benzodiazepine and other painkiller prescriptions, respectively. This translates into a total of 34,520 HZ-related opioid prescriptions, 9,413 prescriptions for benzodiazepines and 22,406 prescriptions for other painkillers that could potentially be prevented through vaccination (Figure 4).

To avoid one opioid prescription, 19 subjects would need to be vaccinated, and 69 and 29 subjects would need to be vaccinated to avoid one benzodiazepine and one other painkiller prescription, respectively. Assuming that 6% of subjects receiving at least one opioid prescription continue on to have long-term opioid use,^31,32^ defined as continued opioid use after one year from initial prescription, RZV vaccination could impede 1,159 subjects from long-term opioid use, which may increase their risk of addiction. The impact of RZV vaccination on opioid-related ED visits avoided (3.3 per million adults aged ≥50 years) and deaths avoided (1.2 per million adults aged ≥50 years) was modest.

## Discussion

Our study shows that opioids are widely used in subjects suffering from an episode of HZ. Even though most subjects received opioids as second-line treatment after prior treatment with an antiviral agent, ≥25% of subjects with opioid prescriptions received opioids as first-line treatment in combination with antivirals. PHN was the single most predictive factor for opioid prescription. Current guidelines recommend opioids as third-line treatment for PHN-related pain after failure of gabapentinoids and tricyclic antidepressants.^6^ Treatment of PHN-related pain is challenging since different pathophysiological mechanisms may be involved in the development of pain; this heterogeneity translates into a high rate of refractoriness to first and second-line therapies and explains the continued need for opioids.^5^

Other factors that were associated with opioid prescription included immunocompromising conditions, comorbidities, geographic region and earlier calendar years of HZ index date. Immunocompromised status and the presence of comorbidities are known risk factors for developing HZ, but the connection with opioid prescription has not been documented in a large patient cohort. Reasons for increased likelihood of opioid prescriptions in these subjects cannot be evaluated based on our study design and needs further examination. This study also found that patients in the Northeast geographic region were significantly less likely to be prescribed opioids compared with patients in other geographic regions. Geographic differences in the number of opioid prescriptions were previously reported. In a cross-sectional study analyzing annual opioid prescriptions from retail, non-hospital pharmacies between 2006 and 2017, an approximately two-fold variation in morphine milligram equivalents per person was observed among states.^33^ Variations in opioid prescription rates across geographic region might be due to differing state legislation with 33 states having put in place regulations regarding opioid use.^34,35^ The observed decrease in opioid prescriptions during later calendar years reflects the general opioid-prescribing pattern in the US, which has plateaued or decreased after 2012, following the launch of several initiatives to regulate opioid prescription to counter abuse, overdose, and opioid-related mortality.^36,37^

Use of gabapentinoids, tricyclic antidepressants and local analgesic patches may help in reducing opioid prescriptions, but these treatments do not offer satisfactory solutions to all patients with HZ/PHN-related pain. Gabapentinoids have their own caveats including a potential risk for abuse,^38^ and patients may be refractory to other painkillers requiring more than one agent to provide acceptable pain relief.^5^ Identifying patients who are at greater risk of addiction after initiation of opioid treatment for pain has proven difficult.^39^ Future interventions should focus on prevention of HZ and PHN, especially in those patients with comorbidities and immunocompromised status.

Our study highlighted that HCRU and total health care costs were higher in the opioid HZ cohort versus the non-opioid HZ cohort or matched non-HZ cohort. This increase was mainly driven by an increase in inpatient stays, ED, and outpatient visits. Reducing the number of opioid prescriptions in the HZ population may therefore have a significant impact on overall health care costs. Based on opioid prescription rates from our claims database analysis and RZV vaccine efficacy data, we estimated that ≥19,000 subjects would avoid opioid prescriptions in a population of 1 million adults aged ≥50 years.

Study limitations are linked to the cross-sectional, retrospective study design. For example, a causal relationship between HZ/PHN-related opioid prescription and long-term opioid use could not be evaluated. However, we could determine the number of subjects with continued opioid prescriptions after one year from the HZ index date (between 6% to 8%), which is in line with other studies.^31, 32^ It was not feasible to ascertain the reason for opioid prescription (e.g., HZ- or non-HZ-related) and opioid prescriptions (especially those in the later months of the 1-year follow-up period) may have been due to other conditions. Our study did not consider potential differences in the risk of abuse between different opioids. However, the type of opioid seems to play a minor role and rather, abuse is driven by a patient's propensity for opioid-induced craving^40^and by the duration and cumulative dose of initial opioid prescription.^31^ While claims data point toward a decrease in opioid prescriptions over the study period, the direct impact of recent opioid prescribing policies on prescription rate has not been analyzed. To what extent the use of opioids in the context of HZ or PHN contributes to the much-debated opioid crisis is unknown. While there is no one solution to counter this crisis, HZ vaccination may contribute to a solution by potentially reducing the number of first opioid prescriptions in people at risk of HZ and PHN.

HZ-related treatment patterns are similar across geographic regions in the US and health care plans. Even though the number of opioid prescriptions has decreased during recent years, opioids will maintain their therapeutic place in the management of HZ-related pain. A preventive strategy using RZV vaccination can potentially be effective in reducing HZ-related opioid prescriptions as well as HRCU and health care costs. A video which summarizes key findings of this manuscript can be found at https://dx.doi.org/10.6084/m9.figshare.19207806.

## Trademark

*Shingrix* is a trademark owned by or licensed to the GSK group of companies.
